# ABRA: improved coding indel detection via assembly-based realignment

**DOI:** 10.1093/bioinformatics/btu376

**Published:** 2014-06-06

**Authors:** Lisle E. Mose, Matthew D. Wilkerson, D. Neil Hayes, Charles M. Perou, Joel S. Parker

**Affiliations:** ^1^Lineberger Comprehensive Cancer Center, ^2^Department of Genetics, ^3^Division of Medical Oncology, Department of Internal Medicine, Multidisciplinary Thoracic Oncology Program, University of North Carolina at Chapel Hill, Chapel Hill, NC 27599, USA

## Abstract

**Motivation:** Variant detection from next-generation sequencing (NGS) data is an increasingly vital aspect of disease diagnosis, treatment and research. Commonly used NGS-variant analysis tools generally rely on accurately mapped short reads to identify somatic variants and germ-line genotypes. Existing NGS read mappers have difficulty accurately mapping short reads containing complex variation (i.e. more than a single base change), thus making identification of such variants difficult or impossible. Insertions and deletions (indels) in particular have been an area of great difficulty. Indels are frequent and can have substantial impact on function, which makes their detection all the more imperative.

**Results:** We present ABRA, an assembly-based realigner, which uses an efficient and flexible localized *de novo* assembly followed by global realignment to more accurately remap reads. This results in enhanced performance for indel detection as well as improved accuracy in variant allele frequency estimation.

**Availability and implementation:** ABRA is implemented in a combination of Java and C/C++ and is freely available for download at https://github.com/mozack/abra.

**Contact:** lmose@unc.edu; parkerjs@email.unc.edu

**Supplementary information:** Supplementary data are available at *Bioinformatics* online.

## 1 INTRODUCTION

A number of realignment or assembly methods have been proposed to overcome the alignment errors and reference bias that limit indel detection. Short read micro aligner locally realigns reads to regionally assembled variant graphs (Homer and Nelson, 2010). Pindel uses a pattern growth approach to detect indels (Ye *et al.*, 2009). Dindel realigns reads to candidate haplotypes and uses a Bayesian method to call indels up to 50 bp in length (Albers *et al.*, 2011). The Genome Analysis Toolkit (GATK)’s IndelRealigner seeks to minimize the number of mismatching bases via local realignment (DePristo *et al.*, 2011). Whole-genome *de novo* assembly approaches include Fermi (Li, 2012) and Cortex Var (Iqbal *et al.*, 2012). SOAPIndel performs localized assembly and calling on regions containing reads where only one half of a paired read is mapped (Li *et al.*, 2012). Clipping REveals STructure (CREST) uses soft clipped reads and localized assembly to identify somatic structural variants (Wang *et al.*, 2010). Targeted Iterative Graph Routing Assembler (TIGRA) uses targeted assembly to produce contigs from putative breakpoints (Chen *et al.*, 2014). Additional proprietary localized assembly methods have been developed by Complete Genomics (Carnevali *et al.*, 2012) and Foundation Medicine (Frampton *et al.*, 2013).

Our newly developed tool called ABRA accepts a Sequence Alignment/Map (SAM/BAM) file as input and produces a realigned BAM file as output, allowing flexibility in selection of variant calling algorithms and other downstream analysis. Global realignment allows reads that are unaligned or improperly mapped to be moved to a correct location. ABRA detects variation that is not present in the original read alignments and improves allele-frequency estimates for variation that is present. ABRA can be used to enhance both germ-line and somatic variant detection and works with paired-end as well as single-end data.

## 2 METHODS

The ABRA algorithm consists of localized region assembly, contig building, alignment of assembled contigs and read realignment.

Localized assembly of reads is done on small genomic regions of size ≤2 kb. For exome or targeted sequencing, these regions roughly correspond to capture targets. For each region, a De Bruijn graph of *k*-mers is assembled from the input reads (Pevzner *et al.*, 2001). *K*-mers containing low quality or ambiguous bases are filtered and *k*-mers that do not appear in at least two distinct reads are pruned from the graph, reducing the impact of sequencing errors on the assembly process.

After initial pruning of the assembled graph, the graph is traversed to build contigs longer than the original read length. There is no smoothing of the graph to remove low-frequency variation, as we are interested in detecting such variation. All non-cyclic paths through the graph are traversed. In cases where a cycle in the graph is observed for a given region, that region is iteratively reassembled using increasing *k*-mer sizes until the cycle no longer exists or a configurable maximum *k*-mer size is reached. As currently implemented, detection of local insertions is limited to less than maximum *k*-mer size. Larger insertions of sequence from another location in the genome are likely to be aligned elsewhere and not included in local assembly, thus limiting detection of insertions as the size approaches read length.

Assembled contigs for all regions are aligned to the reference genome. We currently use BWA MEM (Li, 2013) for contig alignment. Chimerically aligned contigs are combined when appropriate (in cases of longer indels). Redundant sequence as well as sequence not varying from the original reference is removed. The result is used as the basis for an alternate reference.

The original reads are mapped to the alternate reference using a non-gapped alignment. Reads that unambiguously align more closely to the alternate than the original reference are modified to reflect the updated alignment information in the context of the original reference.

Typical ABRA runtime for a human whole exome of depth 150X on a machine with eight cores is roughly 2 h using <16 GB of RAM.

## 3 RESULTS

### 3.1 HapMap trio

**Fig. 1.**
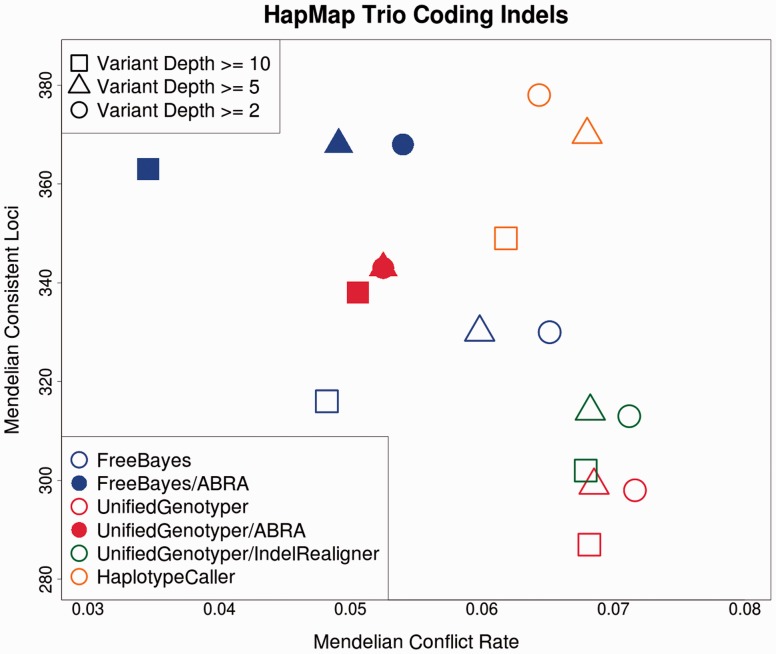
Mendelian consistent loci and Mendelian conflict rates for Freebayes and UnifiedGenotyper both pre- and post-ABRA. UnifiedGenotyper results with GATK Local Realignment around Indels as well as HaplotypeCaller results are also shown for comparison. Shapes in this figure represent variant depth, whereas color/shading represent caller and realignment method

ABRA was applied to exome target regions of a CEPH Hapmap trio of three individuals sequenced to 50x as part of the Illumina Platinum Genomes project and aligned using bwa mem. Variants were called with and without ABRA using Freebayes (Garrison and Marth, 2012) and UnifiedGenotyper (DePristo *et al.*, 2011). The GATK’s HaplotypeCaller was used to call variants without ABRA and the GATK’s IndelRealigner was applied to UnifiedGenotyper input. Coding indels with variant-allele frequency of ≥20% are used in this germ-line evaluation. ABRA enables an increase in the number of Mendelian consistent loci (MCL) detected and a decrease in Mendelian conflict rate (MCR) with either Freebayes or UnifiedGenotyper (Fig. 1). The Freebayes/ABRA combination yields a decrease in MCR compared with HaplotypeCaller and remains competitive in number of MCL detected. Pre-/post-ABRA concordance for Mendelian consistent SNP loci is >99%. Although we anticipate that ABRA will also provide improved performance in non-coding regions, this has not yet been explored.

### 3.2 TCGA tumor and normal data

**Fig. 2.**
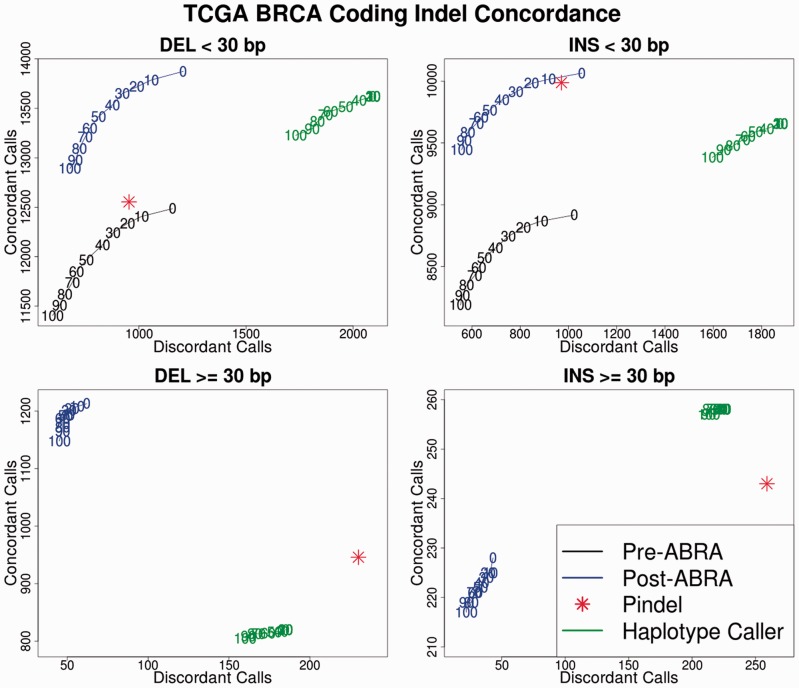
Concordance/discordance with TIGRA assembled contigs for predicted calls from FreeBayes (pre- and post-ABRA), Pindel and Haplotype Caller. Indels within the ranges enabled by ABRA are evaluated (deletions up to 2000 bp and insertions up to the read length). The numbers in the figure represent a cutoff point for variant quality scores as reported in the respective caller’s VCF output. A small number of pre-ABRA deletions >30 bp and 0 pre-ABRA insertions >30 bp are called. FreeBayes currently does not use reads partially overlapping an insert as supporting evidence, which may impact post-ABRA sensitivity for longer insertions

We applied ABRA to 100 normal exomes from the Breast Invasive Carcinoma (BRCA) cohort of The Cancer Genome Atlas (TCGA) project (The Cancer Genome Atlas Network, 2012) using BWA (Li and Durbin, 2009) for the initial alignments. Germ-line variants were called both with and without ABRA using FreeBayes. We also called germ-line variants using HaplotypeCaller and Pindel for comparison purposes. To evaluate these calls in the absence of ground truth, we assembled predicted calls for all methods using TIGRA and aligned the resulting contigs with the BLAST-like alignment tool (BLAT) (Kent *et al.*, 2002). ABRA increased concordance with the TIGRA/BLAT results and maintained a low discordance rate (Fig. 2). Further, ABRA generated estimated allele frequencies closer to 50 and 100%, which is expected in a diploid individual (see Supplementary Material). We next compared pre- and post-ABRA somatic variant calls on 750 TCGA BRCA normal/tumor exome pairs. Strelka (Saunders *et al.*, 2012) and UNCeqR (Wilkerson *et al.*, 2012) were used for somatic calling. Improved detection of somatic mutation was observed in the post-ABRA calls (see Supplementary Material).

## 4 CONCLUSION

ABRA improves on next-generation sequencing read alignments, providing enhanced performance in detection of indels as well as greater accuracy in variant allele frequency estimation. ABRA accepts BAM files as input and outputs realigned BAM files, allowing flexibility in downstream analysis. ABRA can be used with a variety of variant callers for both germ-line and somatic variant calling.

*Funding*: This work was supported in part by the National Cancer Institute Breast SPORE program (P50-CA58223-09A1) and The Cancer Genome Atlas (U24-CA143848-05).

*Conflict of Interest*: none declared.

## Supplementary Material

Supplementary Data
