# Supplementary material for: ABRA: improved coding indel detection via assembly-based realignment
Source: Bioinformatics. 2014 Jun 6;30(19):2813–5. doi: 10.1093/bioinformatics/btu376 (PMC4173014; doi:10.1093/bioinformatics/btu376)
Supplement: Supplementary Data [file supp_30_19_2813__index.html]

ABRA: improved coding indel detection via assembly based re-alignment — ABRA: improved coding indel detection via assembly-based realignment — ABRA: improved coding indel detection via assembly-based realignment — Supplementary Data 

# ABRA: improved coding indel detection via assembly-based realignment

## Supplementary Data

files

**Files in this Data Supplement:**

- Supplementary Data - pdf file
