# Supplementary material for: ABRA: improved coding indel detection via assembly-based realignment
Source: Bioinformatics. 2014 Jun 6;30(19):2813–5. doi: 10.1093/bioinformatics/btu376 (PMC4173014; doi:10.1093/bioinformatics/btu376)
Supplement: Supplementary Data [file supp_btu376_abra_supplementary_material.pdf]

Supplementary Material for ABRA: improved coding indel detection via assembly based re-alignment

TCGA BRCA Somatic Variants

We applied ABRA to 750 BRCA tumor / normal pairs. Tumor and normal samples were assembled together resulting in a single shared alternate reference for both samples. Variants were called on the original BAMs (pre-ABRA) as well as on the ABRA realigned BAMs (post-ABRA) using Strelka. Predicted indels were passed into TIGRA for assembly with the resulting contigs aligned via BLAT. A called indel is considered concordant if the TIGRA / BLAT result shows evidence of the variant in the tumor, but not in the normal. Improved indel detection performance is observed in the post ABRA results. The total number of single nucleotide variants (SNVs) called pre-ABRA is 619,911 versus 617,872 post-ABRA. The figure below reflects results of indel calls using Strelka. The numbers in the figure represent a cutoff point for quality scores reported by Strelka.

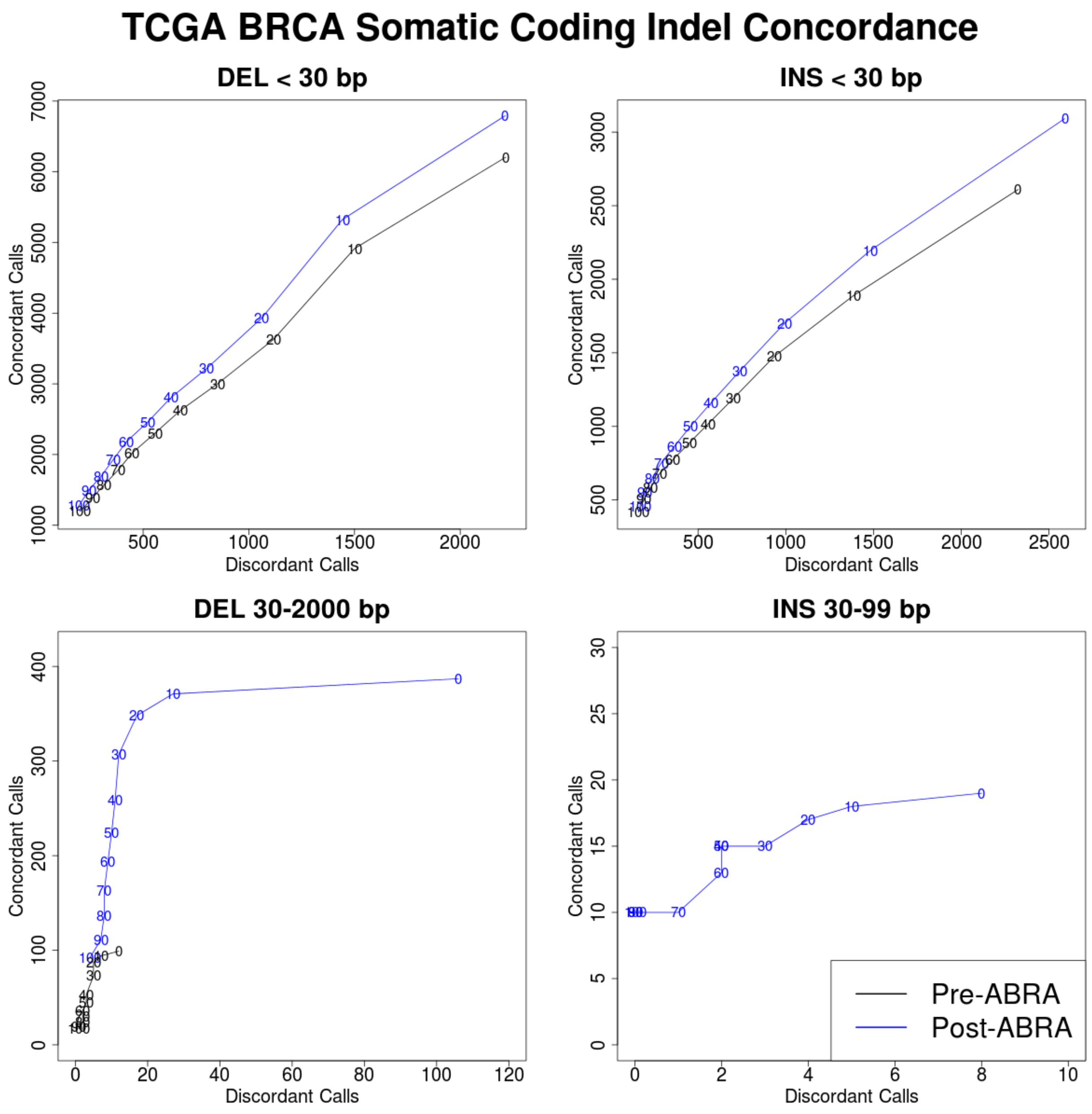

Figure 1. TCGA Somatic Indel Concordance with TIGRA and BLAT

TCGA BRCA Germline Alignment Quality

Noisy read alignments containing disparate gaps, mismatches to the reference or high base quality soft clipping may be indicative of the presence of a more complex variant. An example of noisy pre ABRA alignments (top panel) and more parsimoniously represented post ABRA alignments (bottom panel) is below.

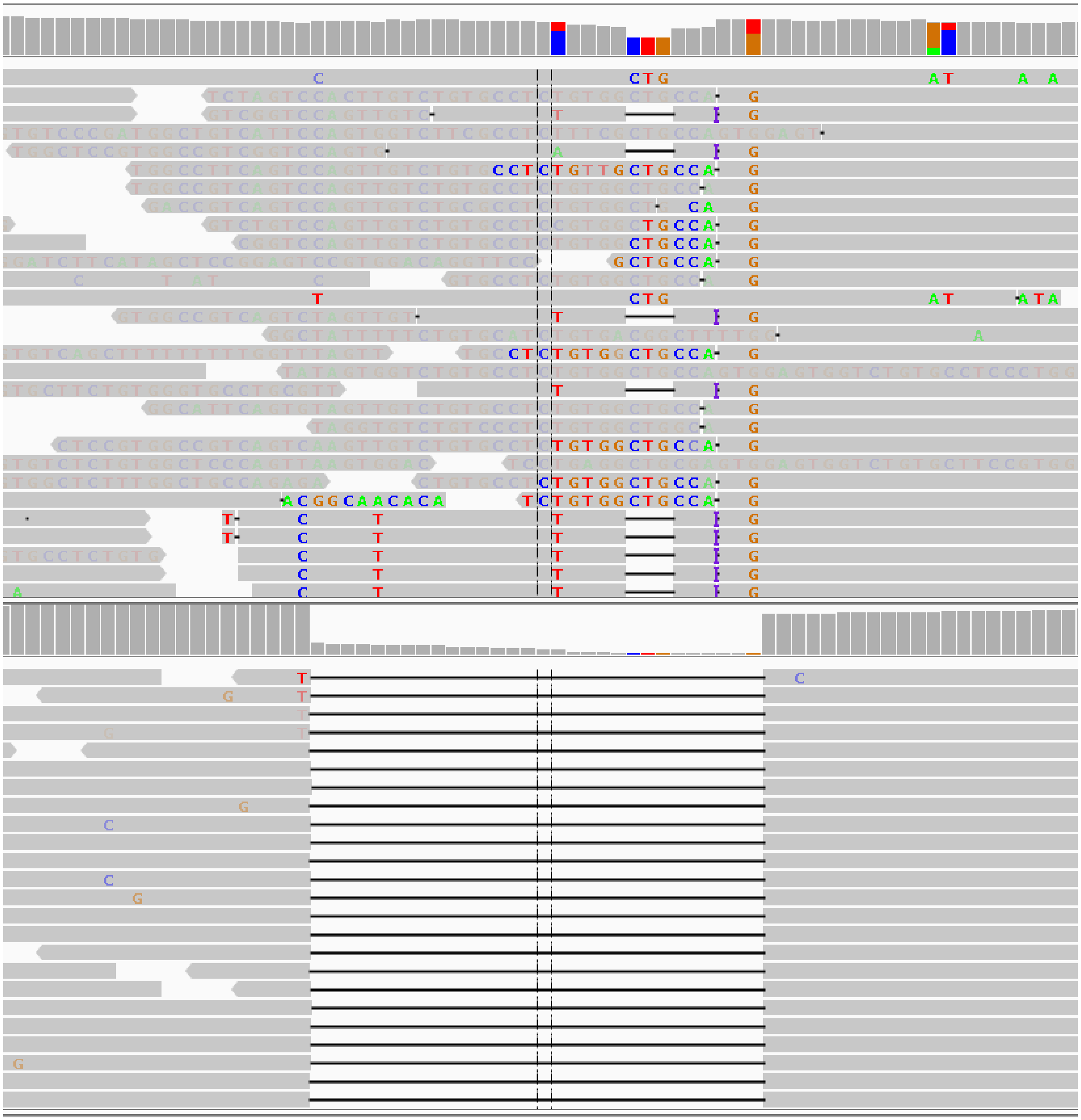

Figure 2. Example of "noisy" alignments pre-ABRA and "cleaned" alignments post-ABRA

We introduce Mismatch Density (MMD) as a measure of quality of alignments for reads near called indels. MMD for a given indel locus is measured by the number of high quality bases not matching the reference genome (inclusive of soft clipped bases) summed with the number of indels (not including the called indel) within a read length of the position of interest. In the 750 sample BRCA cohort, we measure MMD at loci for germline indels called specific to pre-ABRA alignments, post-ABRA alignments and indels called in both sets of alignments (common calls). For each of the 3 sets of calls, alignments in both pre ABRA and post ABRA BAMs are examined. In all 3 groups, MMD is reduced in post ABRA alignments compared to pre ABRA alignments. A total of 6,055,130 simple SNPs (SNPs at least a read length away from another variant) are called pre ABRA and 6,051,391 are called post ABRA.

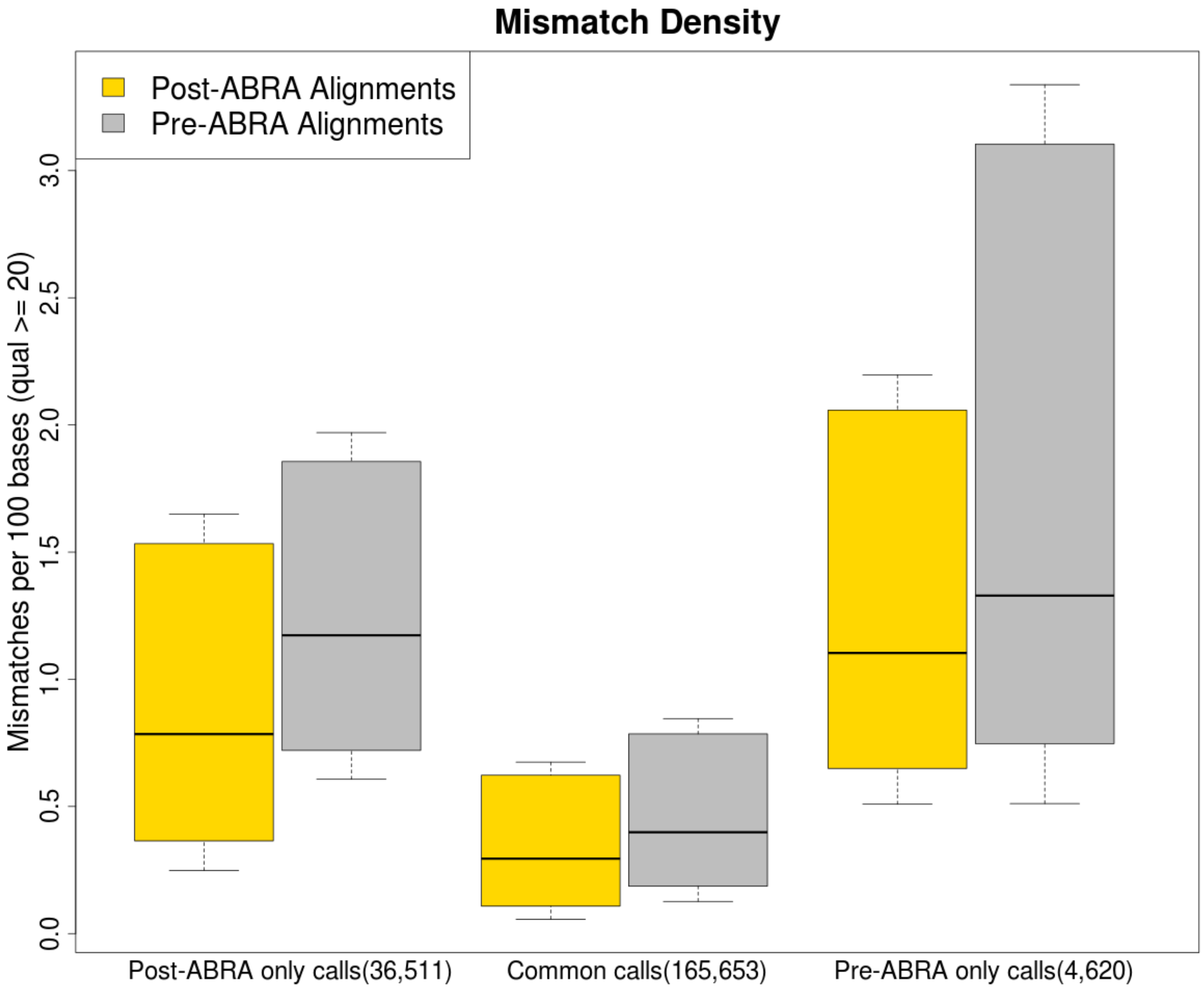

Figure 3. Mismatch Density (MMD) for pre and post-ABRA alignments across 3 sets of calls (pre-ABRA specific, post-ABRA specific and common to both)

TCGA BRCA Germline Allele Frequency

Working from indel variant calls common to both pre and post ABRA results in the BRCA cohort, we compared allele frequency of post-ABRA variant calls with pre-ABRA variant calls. Post-ABRA variant calls generally have allele frequencies closer to the 50% and 100% rates expected in a diploid individual.

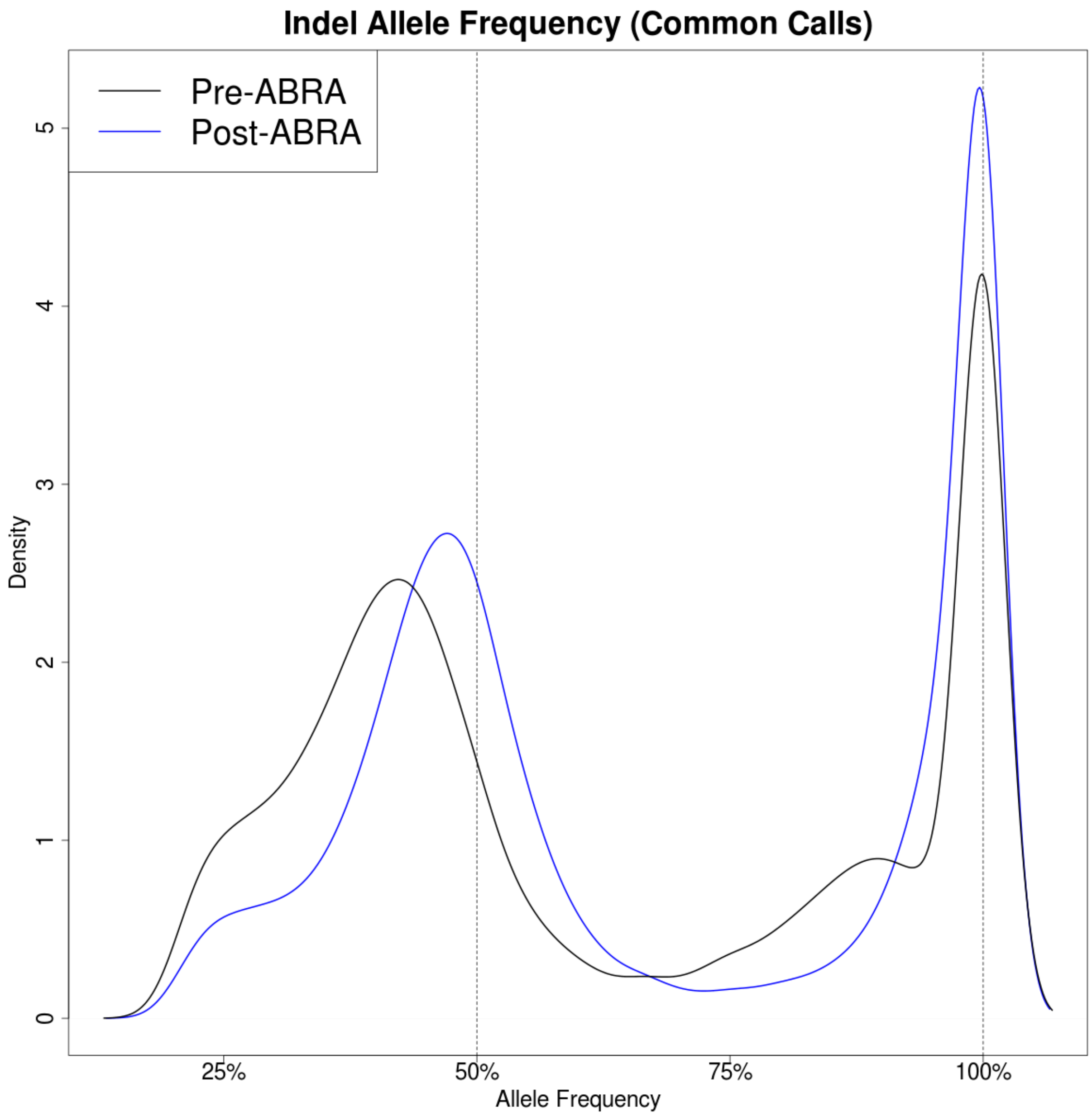

Figure 4. TCGA Germline Allele Frequencies

Simulated Somatic Evaluation

To evaluate ABRA's impact on somatic variant calling, we simulated over 10000 variants in approximately 200 cancer genes of interest. SNVs and indels ranging in length from 1 to 100 bases were spiked into test datasets at frequencies ranging from 5 to 50%. The reads for this simulation are of length 100bp. Initial alignments were performed using BWA-MEM. We compare results of Strelka variant calling with no realignment, GATK Local Alignment around Indels and ABRA realignment. ABRA enables improved performance for indels of length 10 or more. The numbers on the plot represent variant quality scores reported by Strelka.

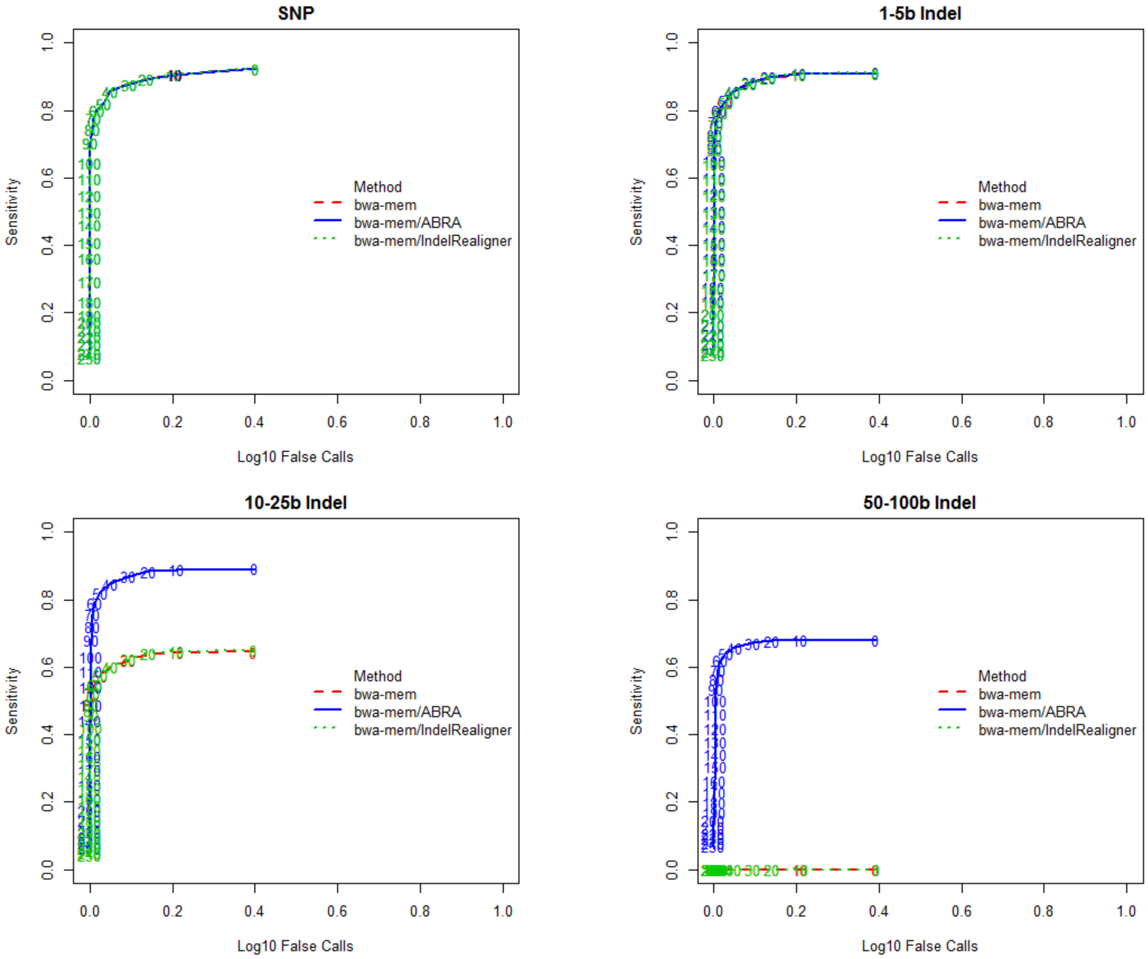

Figure 5. Evaluation of ABRA's somatic variant calling performance on simulated data

In the same simulated dataset, we examine mutant allele frequency (MAF) estimation. ABRA realigned results display improved MAF estimation across all indel lengths.

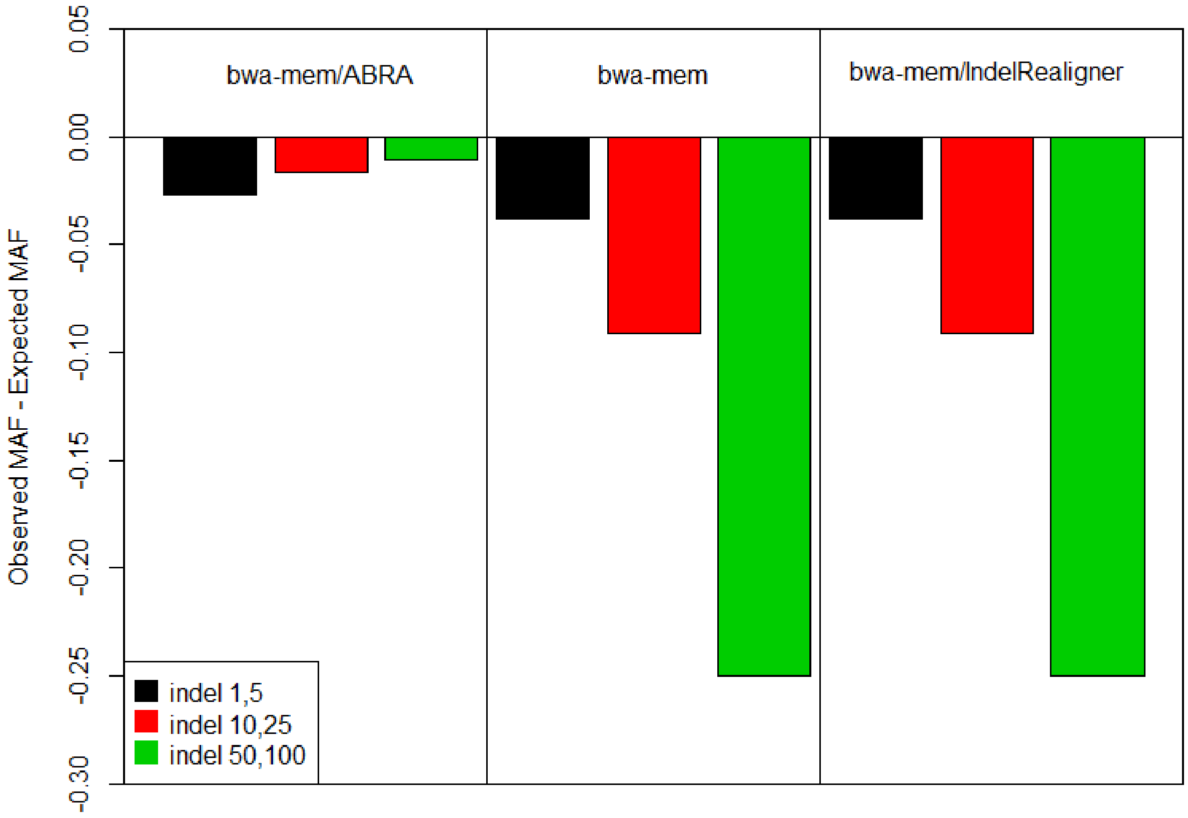

Figure 6. Evaluation of ABRA's somatic mutant allele frequency(MAF) estimation on simulated data
